# Supplementary material for: Herding unmasked: Insights into cryptocurrencies, stocks and US ETFs
Source: PLoS One. 2025 Feb 3;20(2):e0316332. doi: 10.1371/journal.pone.0316332 (PMC11790157; doi:10.1371/journal.pone.0316332)
Supplement: S1 Appendix — (PDF) [file pone.0316332.s001.pdf]

# Supplemental Material

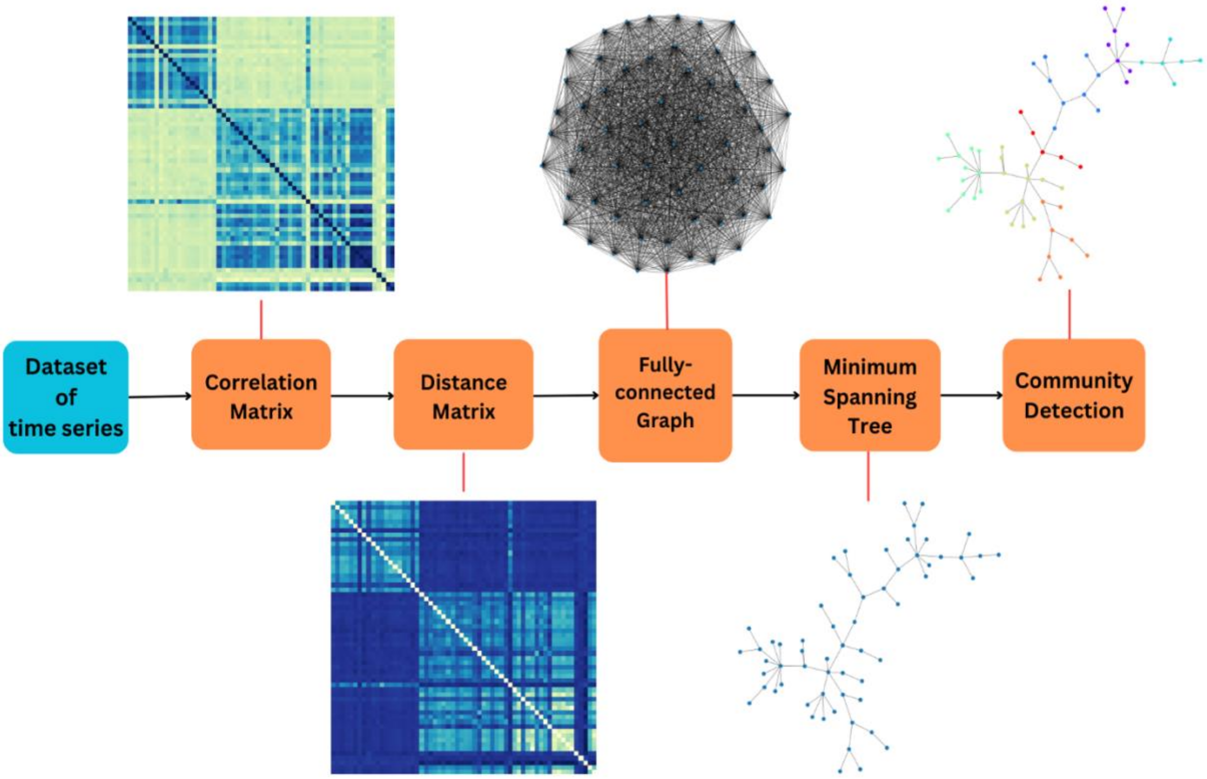


**The process of community detection**

Firstly, a set of time series is determined, where each time series corresponds to an asset. Secondly, a Pearson-based correlation matrix is constructed from the historical time series. the darker the color, the more similar the two corresponding assets. Thirdly, a distance matrix is obtained from the correlation matrix. Fourthly, a fully connected graph is constructed based on the distance between each pair of assets from the previous step. Specifically, although every two arbitrary nodes are connected, their corresponding edge has a different weight which represents the distance between them, the lower the weight, the nearer the two nodes. Fifthly, a minimum spanning tree (MST) is extracted from the distance matrix to reduce the size of the graph while keeping essential information. Lastly, Louvain community detection technique is applied to the MST to detect existing communities in the graph. Each community contains nodes that share similar characteristics and nodes in different communities have different characteristics.
