# Supplementary material for: Herding unmasked: Insights into cryptocurrencies, stocks and US ETFs
Source: PLoS One. 2025 Feb 3;20(2):e0316332. doi: 10.1371/journal.pone.0316332 (PMC11790157; doi:10.1371/journal.pone.0316332)
Supplement: S1 Text — (PDF) [file pone.0316332.s002.pdf]

Supplemental Material

Visualization of herding detection results and sector distribution.

The figure illustrates herding detection results and sector distribution of each community. a) herding results for each community. To visualize these herding coefficients, we use their absolute values and refer to them as *herding magnitudes*, if there is no evidence of herding, the herding magnitude is equal to 0. In this regard, the grey bar means the herding based on CSAD model (Eq 3) is significant, the green bar means the herding based on CSAD for the *up* market condition (Eq 4) is significant, the red bar means the herding based on CSAD for *down* market condition (Eq 4) is significant. The higher the bar, the more pronounced the herding; b) sector distribution (percentage of each sector) of each community, sectors are listed on the y-axis, each represented by a unique color. For each community, the longer the horizontal bar of a corresponding sector, the greater the sector's percentage within that community.
