# Supplementary material for: Herding unmasked: Insights into cryptocurrencies, stocks and US ETFs
Source: PLoS One. 2025 Feb 3;20(2):e0316332. doi: 10.1371/journal.pone.0316332 (PMC11790157; doi:10.1371/journal.pone.0316332)
Supplement: S2 Text — (PDF) [file pone.0316332.s003.pdf]

Supplemental Material

Time-varying community structures of cryptocurrencies, stocks and US ETFs

In each sub-figure, assets with the same color belong to one community. The black-shaped oval highlights an example of a community containing stocks and US ETFs where the centre of the community is a US ETF, which is linked by surrounding stocks. Moreover, this US ETF tends to be the basket of its surrounding stocks, meaning that it is designed to track the overall performance of those stocks. The purple-shaped oval highlights communities containing cryptocurrencies. As can be seen, most cryptocurrencies are close to each other, forming communities among themselves and showing a certain distinction level from stocks and US ETFs.
