# Supplementary material for: Herding unmasked: Insights into cryptocurrencies, stocks and US ETFs
Source: PLoS One. 2025 Feb 3;20(2):e0316332. doi: 10.1371/journal.pone.0316332 (PMC11790157; doi:10.1371/journal.pone.0316332)
Supplement: S1 Table — (PDF) [file pone.0316332.s004.pdf]

Supplemental Material

Three investment vehicles used in this study and their corresponding assets

Classes	Assets
**Cryptocurrencies**	ADA (Cardano), BAT (Basic Attention Token), BCH (Bitcoin Cash), BNT (Bancor), BSV (Bitcoin SV), BTC (Bitcoin), DAI (Dai), DASH (Dash), DOGE (Dogecoin), EOS (EOS), ETH (Ethereum), ETC (Ethereum Classic), LTC (Litecoin), NEO (Neo), OMG (OMG Network), QTUM (Qtum), REP (Augur), TRX (TRON), USDT (Tether), UST (TerraUSD), XLM (Stellar), XMR (Monero), XRP (Ripple), XTZ (Tezos), XVG (Verge), ZEC (Zcash), ZRX (Ox)
**US ETFs**	CIBR (First Trust Nasdaq Cybersecurity ETF), DIA (SPDR Dow Jones Industrial Average ETF), DJD (Invesco Dow Jones Industrial Average Dividend ETF), DJP (iPath Bloomberg Commodity Index Total Return ETN), DVY (iShares Select Dividend ETF), FDLO (Fidelity Low Volatility Factor ETF), FDN (First Trust Dow Jones Internet Index Fund), FNGU (MicroSectors FANG+ Index 3X Leveraged ETN), FTXO (First Trust Nasdaq Bank ETF), IBB (iShares Nasdaq Biotechnology ETF), IDU (iShares U.S. Utilities ETF), IEO (iShares U.S. Oil & Gas Exploration & Production ETF), IJH (iShares Core S&P Mid-Cap ETF), IJJ (iShares S&P Mid-Cap 400 Value ETF), IJK (iShares S&P Mid-Cap 400 Growth ETF), IJR (iShares Core S&P Small-Cap ETF), IJS (iShares S&P Small-Cap 600 Value ETF), IJT (iShares S&P Small-Cap 600 Growth ETF), ITB (iShares U.S. Home Construction ETF), ITOT (iShares Core S&P Total U.S. Stock Market ETF), IUSG (iShares Core S&P U.S. Growth ETF), IVE (iShares S&P 500 Value ETF), IWB (iShares Russell 1000 ETF), IWM (iShares Russell 2000 ETF), IWV (iShares Russell 3000 ETF), IYE (iShares U.S. Energy ETF), IYF (iShares U.S. Financials ETF), IYJ (iShares U.S. Industrials ETF), IYR (iShares U.S. Real Estate ETF), IYT (iShares Transportation Average ETF), IYW (iShares U.S. Technology ETF), IYY (iShares Dow Jones U.S. ETF), KBWB (Invesco KBW Bank ETF), OEF (iShares S&P 100 ETF), ONEQ (Fidelity Nasdaq Composite ETF), PNQI (Invesco Nasdaq Internet ETF), QQQ (Invesco QQQ Trust), QTEC (First Trust NASDAQ-100 Technology Sector Index Fund), QYLD (Global X NASDAQ 100 Covered Call ETF), ROBT (First Trust Nasdaq Artificial Intelligence and Robotics ETF), RPG (Invesco S&P 500 Pure Growth ETF), RPV (Invesco S&P 500 Pure Value ETF), SLYG (SPDR S&P 600 Small Cap Growth ETF), SLYV (SPDR S&P 600 Small Cap Value ETF), SLY (SPDR S&P 600 Small Cap ETF), SOXX (iShares PHLX Semiconductor ETF), SPY (SPDR S&P 500 ETF), SVXY (ProShares Short VIX Short-Term Futures ETF), XTN (SPDR S&P Transportation ETF).
**Stocks**	AAPL (Apple Inc.), ABBV (AbbVie Inc.), ABB (ABB Ltd.), ABT (Abbott Laboratories), ACN (Accenture plc), ADBE (Adobe Inc.), ADI (Analog Devices, Inc.), ADP (Automatic Data Processing, Inc.), AIR (AAR Corp.), ALV (Autoliv Inc.), AMAT (Applied Materials, Inc.), AMD (Advanced Micro Devices, Inc.), AMGN (Amgen Inc.), AMT (American Tower Corporation), AMZN (Amazon.com Inc.), ASML (ASML Holding NV), ASX (ASE Technology Holding Co., Ltd.), AVGO (Broadcom Inc.), AXP (American Express Company), AZN (AstraZeneca PLC), BABA (Alibaba Group Holding Limited), BAC (Bank of America Corp.), BA (The Boeing Company), BDX (Becton, Dickinson and Company), BHP (BHP Group Limited), BKNG (Booking Holdings Inc.), BKR (Baker Hughes Company), BLK (BlackRock Inc.), BMY (Bristol Myers Squibb Company), BP (BP plc), BSX (Boston Scientific Corporation), BTI (British American Tobacco plc), BUD (Anheuser-Busch InBev SA/NV), BX (The Blackstone Group Inc.), CAT (Caterpillar Inc.), CB (Chubb Limited), CFR (Cullen/Frost Bankers, Inc.), CI (Cigna Corporation), CMCSA (Comcast Corporation), CNI (Canadian National Railway Company), COP (ConocoPhillips), COST (Costco Wholesale Corporation), CP (Canadian Pacific Railway Limited), CRM (Salesforce.com Inc.), CSCO (Cisco Systems, Inc.), CSL (Carlisle Companies Incorporated), CVS (CVS Health Corporation), CVX (Chevron Corporation), C (Citigroup Inc.), DEO (Diageo plc), DE (Deere & Company), DHR (Danaher Corporation), DIS (The Walt Disney Company), DTE (DTE Energy Company), ELV (Eaton Vance Corporation), EL (The Estée Lauder Companies Inc.), EOG (EOG Resources Inc.), EQNR (Equinor ASA), ETN (Eaton Corporation plc), GE (General Electric Company), GILD (Gilead Sciences, Inc.), GOOG (Alphabet Inc.), GS (The Goldman Sachs Group, Inc.), HCA (HCA Healthcare, Inc.), HDB (HDFC Bank Limited), HD (The Home Depot, Inc.), HON (Honeywell International Inc.), HSBC (HSBC Holdings plc), IBM (International Business Machines Corporation), IBN (ICICI Bank Limited), IDEX (Ideanomics, Inc.), INTC (Intel Corporation), INTU (Intuit Inc.), ISRG (Intuitive Surgical, Inc.), ITW (Illinois Tool Works Inc.), JNJ (Johnson & Johnson), JPM (JPMorgan Chase & Co.), KO (The Coca-Cola Company), LIN (Linde plc), LLY (Eli Lilly and Company), LMT (Lockheed Martin Corporation), LOW (Lowe's Companies, Inc.), LRCX (Lam Research Corporation), MA (Mastercard Incorporated), MCD (McDonald's Corporation), MC (Moelis & Company), MDLZ (Mondelez International, Inc.), MDT (Medtronic plc), META (Meta Platforms, Inc.), MMC (Marsh & McLennan Companies, Inc.), MO (Altria Group, Inc.), MRK (Merck & Co., Inc.), MSFT (Microsoft Corporation), MS (Morgan Stanley), MUFG (Mitsubishi UFJ Financial Group, Inc.), MU (Micron Technology, Inc.), NEE (NextEra Energy, Inc.), NFLX (Netflix, Inc.), NKE (NIKE, Inc.), NOW (ServiceNow, Inc.), NVDA (NVIDIA Corporation), NVO (Novo Nordisk A/S), NVR (NVR, Inc.), NVS (Novartis AG), ORCL (Oracle Corporation), PANW (Palo Alto Networks, Inc.), PBR (Petroleo Brasileiro S.A. - Petrobras), PDD (Pinduoduo Inc.), PEP (PepsiCo, Inc.), PFE (Pfizer Inc.), PG (Procter & Gamble Co.), PLD (Prologis, Inc.), PM (Philip Morris International Inc.), PYPL (PayPal Holdings, Inc.), QCOM (QUALCOMM Incorporated), SAP (SAP SE), SBUX (Starbucks Corporation), SCHW (The Charles Schwab Corporation), SHEL (Sealed Air Corporation), SHOP (Shopify Inc.), SLB (Schlumberger Limited), SNY (Sanofi), SONY (Sony Group Corporation), SO (The Southern Company), SYK (Stryker Corporation), TD (The Toronto-Dominion Bank), TJX (The TJX Companies, Inc.), TMO (Thermo Fisher Scientific Inc.), TMUS (T-Mobile US, Inc.), TM (Toyota Motor Corporation), TSLA (Tesla, Inc.), TSM (Taiwan Semiconductor Manufacturing Company Limited), TTE (TotalEnergies SE), TXN (Texas Instruments Incorporated), T (AT&T Inc.), UL (Unilever PLC), UNH (UnitedHealth Group Incorporated), UNP (Union Pacific Corporation), UPS (United Parcel Service, Inc.), VRTX (Vertex Pharmaceuticals Incorporated), VZ (Verizon Communications Inc.), V (Visa Inc.), WFC (Wells Fargo & Co.), WMT (Walmart Inc.), XOM (Exxon Mobil Corporation), ZTS (Zoetis Inc.)
