# Supplementary material for: Herding unmasked: Insights into cryptocurrencies, stocks and US ETFs
Source: PLoS One. 2025 Feb 3;20(2):e0316332. doi: 10.1371/journal.pone.0316332 (PMC11790157; doi:10.1371/journal.pone.0316332)
Supplement: S2 Table — (PDF) [file pone.0316332.s005.pdf]

Supplemental Material

Distribution of Stock Sectors in the Stock Dataset. Each Asset in the Dataset is Assigned to an Appropriate Stock Sector.

Information Technology	Healthcare	Financials	Consumer discretionary	Consumer staples	Industrials	Energy	Utilities	Materials	Real estate	Communication services
ASX	ABBV	AXP	ALV	BTI	ABB	BKR	DTE	BHP	AMT	
AAPL	ABT	BAC	AZMN	BUD	AIR	BP	NEE	LIN	PLD	
ACN	AMGN	BLK	BKNG	DEO	BA	COP	SO			
ADBE	AZN	BX	COST	EL	CAT	CVX				
ADI	BDX	CB	CSL	GE	CNI	EQNR				
ADP	BMY	CFR	DIS	HON	CP	PBR				
AMAT	BSX	C	HD	ITW	DE	SHEL				
AMD	CI	GS	IDEX	KO	ETN	SLB				
ASML	CVS	HDB	LOW	MDLZ	LMT	TTE				
AVGO	DHR	HSBC	MCD	MO	UNP	EOG				
BABA	ELV	IBN	NKE	PEP	UPS	XOM				
CMCSA	GILD	JPM	NVR	PG	PYPL					
CRM	HCA	MC	SBUX	PM						
CSCO	ISRG	MMC	TJX	UL						
GOOG	JNJ	MS	TM	WMT						
IBM	LLY	MUFG	TSLA							
INTC	MDT	SCHW								
INTU	MRK	TD								
LRCX	NVO	WFC								
MA	NVS									
META	PFE									
MSFT	SNY									
MU	SYK									
NFLX	TMO									
NOW	UNH									
NVDA	VRTX									
ORCL	ZTS									
PANW										
PDD										
QCOM										
SAP										
SHOP										
SONY										
TMUS										
TSM										
TXN										
T										
VZ										
V										
