# Supplementary material for: Herding unmasked: Insights into cryptocurrencies, stocks and US ETFs
Source: PLoS One. 2025 Feb 3;20(2):e0316332. doi: 10.1371/journal.pone.0316332 (PMC11790157; doi:10.1371/journal.pone.0316332)
Supplement: S3 Table — (PDF) [file pone.0316332.s006.pdf]

Supplemental Material

Assets in each community over the entire period. The red color indicates cryptocurrencies, the blue color indicates US ETFs and the black color indicates stocks

Community	Assets	# Assets
1	AAPL, ADBE, AMGN, GILD, GOOG, INTU, MSFT, SONY, TSLA, VRTX, FNGU, IBB, IYW, ONEQ, QQQ, QYLD	16
2	ABBV, AMT, BMY, BSX, CMCSA, ETN, HCA, HON, IBM, ITW, KO, LLY, MDLZ, MDT, MMC, PEP, PG, PLD, SYK, DJD, IVE, IYJ, IYR	23
3	ABB, ACN, BLK, BX, DIS, EL, ISRG, MA, NKE, ORCL, SAP, SBUX, TMUS, V, ZTS, FDLO, ITOT, IWB, IWV, IYY, SPY, SVXY	22
4	BTI, CAT, CB, DE, DTE, LMT, MO, NEE, PM, SO, T, VZ, DVY, IDU	14
5	ADI, AMAT, AMD, ASML, ASX, AVGO, INTC, LRCX, MU, NVDA, QCOM, TSM, TXN, QTEC, ROBT, SOXX	16
6	ALV, BHP, BKNG, CNI, CP, CSL, MC, NVR, TJX, UNP, UPS, IJH, IJJ, IJK, ITB, IYT, XTN	17
7	ABT, BDX, COST, DHR, TMO, WMT, BNT, BTC, DOGE, ETH, USDT, XVG, IUSG, OEF, RPG	15
8	AIR, IJR, IJS, IJT, IWM, SLYG, SLYV, SLY	8
9	AMZN, BABA, CRM, IDEX, META, NFLX, NOW, PANW, PDD, PYPL, SHOP, CIBR, FDN, PNQI	14
10	ADA, BAT, BCH, BSV, DAI, DASH, EOS, ETC, LTC, NEO, OMG, QTUM, REP, TRX, XLM, XMR, XRP, XTZ, ZEC, ZRX	20
11	ADP, AZN, BA, BUD, CI, CSCO, CVS, DEO, ELV, HD, JNJ, LIN, LOW, MCD, MRK, NVO, NVS, PFE, SNY, TM, UL, UNH, DIA	23
12	AXP, BAC, CFR, C, GS, HDB, HSBC, IBN, JPM, MS, MUFG, SCHW, TD, WFC, FTXO, IYF, KBWB	17
13	BKR, BP, COP, CVX, EOG, EQNR, GE, PBR, SHEL, SLB, TTE, XOM, DJP, IEO, IYE, RPV	16
