# Supplementary material for: Herding unmasked: Insights into cryptocurrencies, stocks and US ETFs
Source: PLoS One. 2025 Feb 3;20(2):e0316332. doi: 10.1371/journal.pone.0316332 (PMC11790157; doi:10.1371/journal.pone.0316332)
Supplement: S4 Table — (PDF) [file pone.0316332.s007.pdf]

Supplemental Material

TABLE 1: Assets in each community during the Pre-Covid-19 sub-period. The red color indicates cryptocurrencies, the blue color indicates US ETFs and the black color indicates stocks

Community	Assets	# Assets
1	ADBE, CRM, GOOG, META, PANW, SHOP, CIBR, FDN	8
2	ABBV, AXP, BUD, CMCSA, COST, HON, LIN, MMC, TJX, WMT, OEF, SPY, SVXY	13
3	ABB, BHP, BLK, CAT, CB, CI, CVS, DE, ELV, ETN, GE, HSBC, ITW, MUFG, TD, UNH, IVE, IYF	18
4	AIR, BX, CNI, CP, CSL, MC, NVR, ORCL, TMUS, UNP, UPS, IJH, IJJ, IJK, ITB, ITOT, IYT, XTN	18
5	ACN, ADP, AMT, DTE, EL, INTU, ISRG, LMT, MA, MCD, NEE, NOW, PLD, PYPL, SAP, SBUX, SONY, SO, V, IDU, IUSG, IYR	22
6	ABT, BDX, BSX, DHR, HDB, IBN, LLY, MDT, MRK, NVS, PFE, SYK, TMO, ZTS	14
7	ADI, AMAT, AMD, ASX, AVGO, INTC, LRCX, MU, NVDA, QCOM, TSM, TXN, SOXX	13
8	AZN, DEO, KO, MDLZ, NVO, PEP, PG, SNY, TM, UL, FDLO, IWB, IWV, IYJ, IYY, RPG	16
9	ALV, BAC, CFR, C, GS, JPM, MS, SCHW, WFC, FTXO, KBWB, RPV	12
10	AMGN, BMY, GILD, HCA, VRTX, IBB, IJR, IJS, IJT, IWM, SLYG, SLYV, SLY	13
11	ADA, BCH, BSV, BTC, DOGE, EOS, ETC, LTC, REP	9
12	BAT, BNT, DAI, DASH, ETH, OMG, USDT, UST, XLM, XMR, XRP, XVG, ZEC, ZRX	14
13	AAPL, AMZN, ASML, BABA, BKNG, CSCO, IDEX, MSFT, NFLX, PDD, TSLA, FNGU, IYW, ONEQ, PNQI, QQQ, QTEC, QYLD, ROBT	20
14	BA, DIS, HD, IBM, JNJ, LOW, NKE, DIA, DJD	9
15	BKR, BP, COP, CVX, EOG, EQNR, PBR, SHEL, SLB, TTE, XOM, DJP, IEO, IYE	14
16	BTI, MO, PM, T, VZ, NEO, QTUM, TRX, XTZ, DVY	10

TABLE 2: Assets in each community during the Covid-19 Pandemic sub-period. The red color indicates cryptocurrencies, the blue color indicates US ETFs and the black color indicates stocks

Community	Assets	# Assets
1	BTI, CB, DE, DTE, ETN, MO, NEE, SO, T, VZ, DVY, IDU	12
2	ABBV, ABT, AZN, BDX, CSCO, DHR, IDEX, JNJ, MA, MRK, NVO, NVS, ORCL, PFE, SNY, TMO, V, IUSG, OEF, ONEQ, SVXY	21
3	ABB, BUD, CI, DEO, DIS, ELV, EL, HD, LOW, MCD, NKE, SBUX, UNH, DIA	14
4	ACN, BLK, BMY, ISRG, LIN, LLY, MDLZ, MDT, MMC, PEP, PG, SAP, SYK, UL, ZTS, USDT, UST, FDLO, ITOT, IWB, IWV, IYY, SPY	24
5	ADI, AMAT, AMD, ASML, ASX, AVGO, LRCX, MU, NVDA, QCOM, TSM, TXN, SOXX	13
6	ADP, BSX, CAT, CMCSA, CNI, CP, CVS, GE, HON, IBM, ITW, KO, LMT, PM, UNP, UPS, DJD, IVE, IYJ, IYT, XTN	21
7	BABA, CRM, META, NFLX, PANW, PDD, SHOP, TSLA, CIBR, FDN, FNGU, PNQI	12
8	ALV, BA, BHP, BKR, BP, COP, CVX, EOG, EQNR, MUFG, PBR, SHEL, SLB, TD, TTE, XOM, DJP, IEO, IYE, RPV	21
9	AMT, AXP, BAC, BX, CFR, C, GS, HDB, HSBC, IBN, JPM, MS, PLD, SCHW, TJX, WFC, FTXO, IYF, IYR, KBWB	21
10	AAPL, ADBE, AMGN, AMZN, COST, GILD, GOOG, INTC, MSFT, PYPL, SONY, TMUS, VRTX, WMT, IBB, QQQ, QYLD	17
11	INTU, NOW, ADA, BAT, BNT, ETH, NEO, OMG, QTUM, XMR, XTZ, XVG, ZRX, IYW, QTEC, RPG	16
12	AIR, BKNG, CSL, HCA, MC, NVR, TM, IJH, IJJ, IJK, IJR, IJS, IJT, ITB, IWM, ROBT, SLYG, SLYV, SLY	19
13	BCH, BSV, BTC, DAI, DASH, DOGE, EOS, ETC, LTC, REP, TRX, XLM, XRP, ZEC	14

TABLE 3: Assets in each community during the first Bull Time sub-period. The red color indicates cryptocurrencies, the blue color indicates US ETFs and the black color indicates stocks

Community	Assets	# Assets
1	AAPL, ADI, AMAT, AMD, ASML, ASX, AVGO, INTC, INTU, LRCX, MSFT, MU, NVDA, QCOM, SONY, TSM, TXN, IYW, QTEC, SOXX	20
2	ABBV, AZN, BMY, JNJ, LLY, MRK, NVO, NVS, PFE, SNY	10
3	ABB, CI, DIS, ELV, EL, LIN, MCD, NKE, SAP, SBUX, UNH, DIA	12
4	ABT, ACN, BDX, BLK, BX, DHR, HD, ISRG, LOW, MA, ORCL, TMO, V, ZTS, FDLO, ITOT, IWB, IWV, IYY, SPY, SVXY	21
5	ADBE, CRM, META, NFLX, NOW, PANW, PDD, PYPL, SHOP, CIBR, FDN, PNQI	12
6	ADP, BA, BSX, BTI, CAT, CMCSA, CSCO, CVS, DE, ETN, HCA, HDB, HON, IBM, IBN, ITW, KO, LMT, MDLZ, MDT, MMC, MO, PEP, PG, PM, SYK, TJX, DJD, IVE	28
7	AIR, ALV, CSL, GE, MC, MUFG, NVR, TM, IJH, IJJ, IJK, IJR, IJS, IJT, ITB, IWM, RPV, SLYG, SLYV, SLY	20
8	BAT, BCH, BSV, DASH, ETC, NEO, OMG, QTUM, REP, XMR, XTZ, ZEC, ZRX	13
9	AMGN, AMZN, BABA, COST, GILD, GOOG, TMUS, TSLA, VRTX, WMT, FNGU, IBB, IUSG, OEF, ONEQ, QQQ, QYLD, ROBT, RPG	19
10	AMT, AXP, BKNG, GS, MS, PLD, IYF, IYR	8
11	BAC, CB, CFR, C, DTE, HSBC, JPM, NEE, SCHW, SO, TD, T, VZ, WFC, DAI, DVY, FTXO, IDU, KBWB	19
12	BHP, BKR, BP, BUD, COP, CVX, DEO, EOG, EQNR, PBR, SHEL, SLB, TTE, UL, XOM, DJP, IEO, IYE	18
13	CNI, CP, UNP, UPS, IYJ, IYT, XTN	7
14	IDEX, ADA, BNT, BTC, DOGE, EOS, ETH, LTC, TRX, USDT, UST, XLM, XRP, XVG	14

TABLE 4: Assets in each community during the second Bull Time sub-period. The red color indicates cryptocurrencies, the blue color indicates US ETFs and the black color indicates stocks

Community	Assets	# Assets
1	AAPL, ADBE, AMZN, GOOG, INTU, META, MSFT, NOW, NVDA, TSLA, VRTX, IBB, IYW, QQQ, QTEC, QYLD, ROBT, RPG	17
2	BLK, BSX, BUD, CSCO, DEO, DIS, LIN, MDT, NKE, SYK, DIA, IYJ	12
3	BAC, BTI, CAT, CFR, C, DE, GE, GS, JPM, MO, MS, MUFG, SCHW, WFC, DVY, FTXO, KBWB, RPV	17
4	ABB, ACN, ADP, BX, CMCSA, EL, HDB, IBN, MA, MCD, SBUX, SONY, V, UST, ITOT, IWB, IWV, IYY, SPY, SVXY	20
5	AMT, AXP, CB, CI, CVS, ELV, ETN, HCA, HON, IBM, ITW, LMT, MMC, ORCL, PLD, TD, TJX, TM, UNH, DJD, IVE, IYF, IYR	22
6	ADI, AMAT, AMD, ASML, ASX, AVGO, INTC, LRCX, MU, QCOM, TSM, TXN, SOXX	13
7	COST, DTE, ISRG, NEE, SAP, SO, TMUS, WMT, DAI, FDLO, IDU, IUSG, OEF, ONEQ	14
8	ALV, BA, BKNG, CNI, CP, CSL, HD, LOW, MC, NVR, UNP, UPS, IJH, IJJ, IJK, ITB, IYT, XTN	17
9	ABBV, ABT, AMGN, AZN, BDX, BMY, DHR, GILD, JNJ, LLY, MRK, NVO, NVS, PFE, SNY, TMO, ZTS	17
10	HSBC, ADA, BCH, BSV, DASH, DOGE, EOS, ETC, NEO, QTUM, REP, TRX, USDT, XLM, XMR, XRP, ZEC	17
11	AIR, IDEX, FNGU, IJR, IJS, IJT, IWM, SLYG, SLYV, SLY	10
12	BABA, CRM, NFLX, PANW, PDD, PYPL, SHOP, CIBR, FDN, PNQI	10
13	KO, MDLZ, PEP, PG, PM, T, UL, VZ	8
14	BHP, BKR, BP, COP, CVX, EOG, EQNR, PBR, SHEL, SLB, TTE, XOM, DJP, IEO, IYE	15
15	BAT, BNT, BTC, ETH, LTC, OMG, XTZ, XVG, ZRX	9

TABLE 5: Assets in each community during the third Bull Time sub-period. The red color indicates cryptocurrencies, the blue color indicates US ETFs and the black color indicates stocks

Community	Assets	# Assets
1	AAPL, ADBE, BABA, COST, GOOG, INTU, MSFT, NFLX, PDD, TSLA, WMT, FNGU, IUSG, IYW, OEF, QQQ, QTEC, QYLD	18
2	CI, CSCO, DEO, DIS, ELV, LIN, MCD, SBUX, TJX, TM, UNH, DIA	12
3	CB, CMCSA, CSL, ETN, GE, HCA, HON, ITW, MA, TMUS, V, DJD, IVE, IYJ	14
4	ABT, BDX, DHR, ISRG, NVO, TMO, VRTX, ZTS, UST, IBB, ROBT, RPG	12
5	ADI, AMAT, AMD, ASML, ASX, AVGO, INTC, LRCX, MU, NVDA, ORCL, QCOM, TSM, TXN, USDT, SOXX	16
6	ADP, BX, EL, HDB, IBN, NKE, FDLO, IJK, ITOT, IWB, IWV, IYY	12
7	ADA, DASH, DOGE, EOS, ETC, NEO, OMG, QTUM, REP, TRX, XMR, XTZ, ZEC	13
8	AIR, BA, IDEX, MC, IJR, IJS, IJT, IWM, SLYG, SLYV, SLY	11
9	ABBV, AMGN, AZN, BMY, GILD, JNJ, LLY, MRK, NVS, PFE, SNY, BAT, ZRX	13
10	AMT, DTE, KO, MDLZ, NEE, PEP, PG, PLD, SO, UL, IDU, IYR	12
11	AMZN, CRM, META, NOW, PANW, PYPL, SHOP, SONY, CIBR, FDN, ONEQ, PNQI	12
12	AXP, BAC, CFR, C, GS, HSBC, JPM, MS, MUFG, SCHW, TD, WFC, FTXO, IYF, KBWB	15
13	BCH, BNT, BSV, BTC, ETH, LTC, XLM, XRP, XVG	9
14	ALV, BHP, BKNG, CNI, CP, HD, LOW, NVR, UNP, UPS, IJH, IJJ, ITB, IYT, XTN	15
15	BKR, BP, COP, CVX, EOG, EQNR, PBR, SHEL, SLB, TTE, XOM, DAI, DJP, IEO, IYE	15
16	BTI, BUD, CAT, CVS, DE, IBM, LMT, MO, PM, T, VZ, DVY, RPV	13
17	ABB, ACN, BLK, BSX, MDT, MMC, SAP, SYK, SPY, SVXY	10

TABLE 6: Assets in each community during the first Ukraine-Russia Conflict sub-period. The red color indicates cryptocurrencies, the blue color indicates US ETFs and the black color indicates stocks

Community	Assets	# Assets
1	AAPL, ACN, ADP, AMZN, BABA, COST, GOOG, MMC, MSFT, NFLX, ORCL, PDD, TMUS, TSLA, WMT, FDLO, FNGU, IUSG, OEF, ONEQ, QQQ, QYLD, RPG, SPY	24
2	BKR, COP, CVX, EOG, EQNR, PBR, SLB, XOM, DJP, IEO, IYE	11
3	ABB, ALV, BA, BKNG, BX, CSL, GE, HD, LOW, MC, NVR, TJX, IJH, IJJ, IJK, ITB	15
4	BLK, BSX, EL, ISRG, MDT, NKE, SAP, SBUX, SONY, SYK, DAI, ITOT, IWB, IWV, IYY, SVXY	16
5	ABT, BDX, CI, CMCSA, CSCO, CVS, DEO, DHR, ELV, HON, LIN, MCD, TMO, UL, UNH, ZTS, DIA	17
6	CNI, CP, ETN, ITW, MA, UNP, UPS, V, IYJ, IYT, XTN	11
7	ADBE, ADI, AMAT, AMD, ASML, ASX, AVGO, INTC, INTU, LRCX, MU, NVDA, QCOM, TSM, TXN, IYW, QTEC, ROBT, SOXX	18
8	AIR, AMGN, GILD, VRTX, IBB, IJR, IJS, IJT, IWM, SLYG, SLYV, SLY	12
9	CRM, DIS, IDEX, META, NOW, PANW, PYPL, SHOP, CIBR, FDN, PNQI	11
10	ABBV, AMT, AZN, BMY, HCA, JNJ, KO, LLY, MDLZ, MRK, NVO, NVS, PEP, PFE, PG, PLD, SNY, DJD, IVE, IYR	20
11	BAC, BTI, CFR, C, HSBC, JPM, MO, PM, WFC, FTXO, KBWB	11
12	BHP, BP, CAT, CB, DE, DTE, IBM, LMT, NEE, SHEL, SO, TTE, T, VZ, DVY, IDU, RPV	17
13	AXP, BUD, GS, HDB, IBN, MS, MUFG, SCHW, TD, TM, ADA, BAT, BNT, BTC, ETH, XLM, XMR, XRP, IYF	18
14	BCH, BSV, DASH, DOGE, EOS, ETC, LTC, NEO, OMG, QTUM, REP, TRX, USDT, XTZ, XVG, ZEC, ZRX	17

TABLE 7: Assets in each community during the second Ukraine-Russia Conflict sub-period. The red color indicates cryptocurrencies, the blue color indicates US ETFs and the black color indicates stocks

Community	Assets	# Assets
1	AAPL, ADBE, BABA, GOOG, IDEX, INTU, META, MSFT, NFLX, PDD, TSLA, FNGU, IYW, ONEQ, QQQ, QTEC, ROBT	17
2	BA, BTI, BUD, CI, COST, CSCO, CVS, DEO, ELV, ETN, HON, IBM, LIN, LMT, MCD, TJX, UL, UNH, WMT, DIA, DJD	21
3	ABB, ALV, BHP, CNI, CP, CSL, HCA, HD, LOW, NVR, UNP, UPS, IJH, IJJ, IJK, ITB, IYT, RPG, RPV, XTN	20
4	ABT, ACN, BDX, BSX, ISRG, MDT, SYK, SPY	8
5	ADI, AMAT, AMD, ASML, ASX, AVGO, INTC, LRCX, MU, NVDA, QCOM, TSM, TXN, SOXX	14
6	ADP, BLK, DHR, TMO, IUSG, IWB, OEF, QYLD	8
7	AIR, MC, IJR, IJS, IJT, IWM, SLYG, SLYV, SLY	9
8	AMGN, AZN, GILD, NVO, NVS, SNY, VRTX, IBB	8
9	AMT, CAT, CMCSA, DE, DTE, GE, ITW, MMC, NEE, ORCL, PLD, SO, TMUS, T, VZ, DVY, IDU, IVE, IYR, SVXY	20
10	AMZN, CRM, NOW, PANW, PYPL, SHOP, CIBR, FDN, PNQI	9
11	AXP, BAC, CB, CFR, C, GS, HDB, HSBC, IBN, JPM, MS, MUFG, SCHW, TD, WFC, DAI, FTXO, IYF, KBWB	19
12	ABBV, BMY, JNJ, KO, LLY, MDLZ, MO, MRK, PEP, PFE, PG, PM	12
13	DIS, ADA, BCH, BNT, BSV, BTC, DASH, DOGE, EOS, ETC, ETH, LTC, TRX, USDT, XLM, XMR, XRP, XVG, ZEC	19
14	BKR, BP, COP, CVX, EOG, EQNR, PBR, SHEL, SLB, TTE, XOM, DJP, IEO, IYE	14
15	BKNG, BX, EL, MA, NKE, SAP, SBUX, SONY, TM, V, ZTS, FDLO, ITOT, IWV, IYJ, IYY	16
16	BAT, NEO, OMG, QTUM, REP, XTZ, ZRX	7
